# ZnO electrocatalyst integrated onto carbon paper for efficient non-aqueous Li–O_2_ batteries

**DOI:** 10.1039/d5ra03545g

**Published:** 2025-08-11

**Authors:** Inhan Kang, Sehun Kim, Su Min Lee, Se Hyun Jeong, Hyeong Ju Ki, Ui Jin Lee, Jae Hyung Ko, Byung Jun Son, Sungjin Kim, Jungwon Kang

**Affiliations:** a Department of Advanced Materials Science and Engineering, Mokpo National University 61 Dorim-ri, 1666 Yeongsan-ro, Cheonggye-myeon, Muan-gun 58554 Jeonnam South Korea ksj840711@gmail.com jwkang17@mokpo.ac.kr

## Abstract

The development of high-performance lithium–oxygen (Li–O_2_) batteries is hindered by challenges including high overpotential and limited cycle life. In this paper, we report the cost-effective and scalable synthesis of a ZnO electrocatalyst directly integrated onto carbon paper *via* a simple dipping and thermal treatment method. The resulting ZnO-on-carbon composite (ZnO on P50) was employed as the cathode in a non-aqueous Li–O_2_ battery. Comprehensive physicochemical characterizations, including XPS, FE-SEM, and TEM, confirmed the successful incorporation and uniform dispersion of ZnO nanoparticles within the carbon matrix. Electrochemical evaluations revealed that the ZnO-based cathode reduced overpotential during charge–discharge cycles and improved energy efficiency by approximately 6.3% over 40 cycles compared to pristine carbon paper (P50). *Ex situ* XRD and SEM analyses further validated the formation and decomposition of Li_2_O_2_ during cycling and suggested enhanced reaction uniformity owing to the catalytic role of ZnO. These findings demonstrate that *in situ*-grown ZnO offers a promising, low-cost strategy for improving the energy efficiency of Li–O_2_ batteries.

## Abstract 2

Electrochemically catalytic composite cathode with ZnO and carbon paper for Li–O_2_ battery.

## Graphical abstract

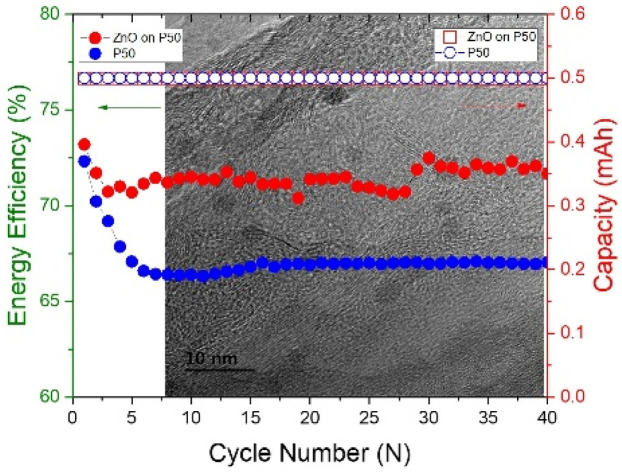

## Introduction

Lithium–oxygen (Li–O_2_) batteries have garnered considerable attention owing to their exceptionally high theoretical energy density, surpassing that of conventional energy storage systems.^1,2^ Among the four types (aprotic, aqueous, all-solid-state, and hybrid aqueous/aprotic) of Li–O_2_ batteries,^3^ rechargeable aprotic Li–O_2_ batteries typically consist of a lithium metal anode, non-aqueous Li^+^-conducting electrolyte, and a porous cathode. In a typical discharge reaction, oxygen undergoes reduction and reacts with lithium ions to form lithium peroxide (Li_2_O_2_), which primarily accumulates on the surface of the air electrode, and the reverse process occurs during charging: 2Li^+^ + O_2_ + 2e^−^ ↔ Li_2_O_2_ (*E*^0^ = 2.96 V, *vs.* Li/Li^+^).^4,5^ Despite these promising features, several important technical challenges, including electrolyte degradation, safety issues related to lithium metal anodes, and high overpotentials (*i.e.*, the voltage gap between charge and discharge), must be addressed to enable the commercialization of Li–O_2_ batteries. One of the main obstacles in developing non-aqueous Li–O_2_ batteries is the large polarization during discharge, and especially during charge (>1.0 V), due to the formation and decomposition of the insulating discharge product, Li_2_O_2_. This high polarization results in low energy efficiency and poor cycling stability.^6,7^

To address these limitations, considerable efforts have focused on incorporating electrocatalysts, such as metals and metal oxides, into carbon-based materials.^8^ However, the use of certain catalysts poses challenges, such as the reliance on expensive precious metals (*e.g.*, Pt, Ru, Au, and Ir)^9–12^ and the complex synthesis routes required for some metal oxides (*e.g.*, CuCo_2_O_4_ and NiCo_2_O_4_).^13,14^ Several studies have investigated ZnO-based catalysts for non-aqueous Li–O_2_ batteries. Luo *et al.* reported that Pd nanoparticles uniformly dispersed on ZnO-passivated porous carbon, synthesized *via* atomic layer deposition, functioned effectively as a cathode material, showing high catalytic activity, particularly for the oxygen evolution reaction.^15^ Yin *et al.* developed hierarchical mesoporous ZnO/ZnFe_2_O_4_/C nanocages, derived from MOF templates, which exhibited a high discharge capacity (>11 000 mAh g^−1^ at 300 mA g^−1^) and improved cyclability performance (5000 mAh g^−1^ over 15 cycles) when used as a Li–O_2_ battery cathode.^16^ However, these approaches often require costly noble metals or involve complex synthetic procedures. Dai *et al.* synthesized CoMn_2_O_4_ using a solvothermal reaction and a polystyrene template and achieved 286 cycles at a current density of 200 mA g^−1^. This was deposited in the form of a film of Li_2_O_2_ due to the enhanced adsorption capacity of the LiO_2_ intermediate, and the DFT results proved that oxygen vacancies are advantageous for LiO_2_ adsorption.^17^ Lin *et al.* achieved 272 cycles at 200 mA g^−1^ by forming low-crystalline Co-oxide and atomic-level dispersion of Ru (double-solvent method) through MOF-based synthesis using ZIF precursor. They reported that low-crystalline cobalt oxide has adsorption properties that promote the formation of film-like LiO_2_ intermediate, and when ruthenium is dispersed at the atomic level, the ORR/OER catalytic activity and Li_2_O_2_ decomposition efficiency are significantly increased.^18^

In this study, we demonstrated the direct *in situ* incorporation of ZnO on carbon paper using a simple dipping and thermal treatment method and evaluated its application in non-aqueous Li–O_2_ batteries. The resulting rechargeable Li–O_2_ cell, employing a ZnO electrocatalyst-integrated carbon paper cathode, exhibited an average ∼6.3% reduction in charge–discharge overpotential over 40 cycles.

## Experimental

### Preparation of materials

Zinc acetate dihydrate (Zn(CH_3_COO)_2_·2H_2_O, Daejung) was dissolved in ethylene glycol (Daejung). Carbon paper (P50, AvCarb®) was immersed in the solution in an amount of 0.01 mol and heated at 400 °C in air for 10 h. After heating, the ZnO electrocatalyst-integrated carbon paper was punched to 12 mm and dried overnight at 120 °C in a vacuum oven.

The moisture in *N,N*-dimethylacetamide (DMAc, Sigma-Aldrich), used as the electrolyte, was removed using activated molecular sieves with a pore size of 4 Å. Lithium nitrate (LiNO_3_, Sigma-Aldrich) was also dried overnight at 120 °C in a vacuum oven. LiNO_3_ was added to DMAc in an amount of 1 mol.

## Characterization of materials

X-ray diffraction (XRD) patterns were recorded using an EMPYREAN/PANalytical multipurpose X-ray diffractometer. X-ray photoelectron spectroscopy (XPS) was carried out using an Al-Kα X-ray source (Multilab 2000, Thermo VG Scientific). The morphology and elemental distribution were examined by field-emission scanning electron microscopy (FE-SEM; S-4800, Hitachi) at 15 kV and field-emission transmission electron microscopy (FE-TEM; Tecnai F20, Philips) at 200 kV.

## Fabrication of Li–O_2_ cells

Swagelok-type cells were assembled in a glove box (MBraun; H_2_O < 0.1 ppm, O_2_ < 1 ppm). Each cell composed a lithium metal anode (Honjo Metal Co), a separator GF (Whatmann™), a carbon cathode (P50, AvCarb®), an electrolyte (DMAc-LiNO_3_), a mesh current collector, and an O-ring. The mass loading of the active material was 6.5 mg, and 0.65 μg of electrolyte was used in each cell.

After assembly, high-purity oxygen gas (>99.995%) was introduced into the inlet and outlet tubes of the cells. An oxygen environment of approximately 1.5 bar was maintained during the charge–discharge cycling by keeping both inlet and outlet valves open. The electrochemical properties of the assembled Li–O_2_ cells were analyzed using a VMP3 potentiostat.

## Results and discussion

A ZnO electrocatalyst integrated onto carbon paper (ZnO on P50) was fabricated using a simple dipping and heating process, as described in the experimental section. Various electrocatalysts for Li–O_2_ batteries have been developed in our laboratory using this method, including Mn_3_O_4_,^19^ NiO,^20^ CuO,^21^ Fe_2_O_3_,^22^ and CaCO_3_.^23^ In most cases, XRD peaks were not clearly observed, likely because of the extremely small amounts of catalyst produced during synthesis. Similarly, the ZnO on P50 in this study did not exhibit any distinguishable ZnO peaks in the XRD patterns when compared with pristine P50, as shown in Fig. S1. Fig. S2 presents the XRD patterns of ZnO and ZnO powder prepared by heating zinc acetate monohydrate at 400 °C in air. This is consistent with the results in the paper by other reports.^24–26^ This observation is consistent with previous findings on electrocatalysts from our laboratory: the ZnO content in the ZnO electrocatalyst-integrated carbon was insufficient for detection by XRD. Therefore, more sensitive surface analysis techniques, such as XPS, are necessary to confirm the presence of ZnO.

**Fig. 1:**
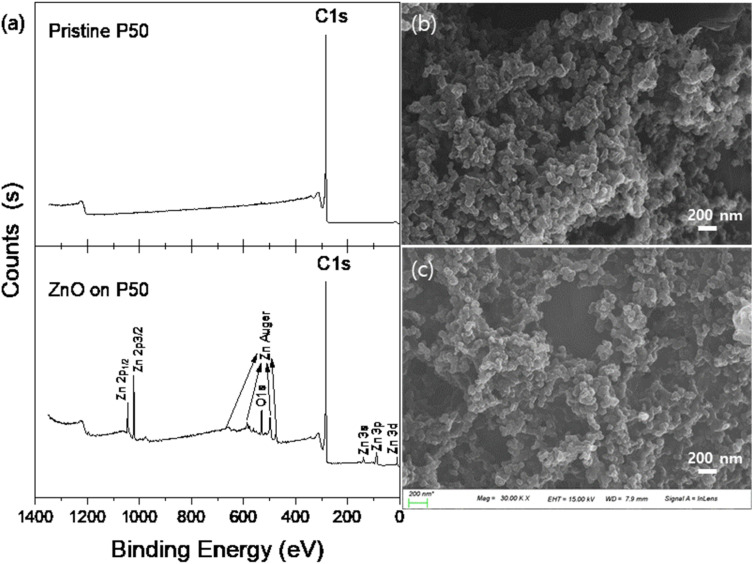
XPS survey scan (a). FE-SEM images of the pristine P50 (b) and ZnO on P50 (c).

Fig. 1 shows the XPS survey scan (a) and FE-SEM images of pristine P50 (b) and ZnO on P50 (c). When comparing pristine P50 (Fig. 1b) and ZnO on P50 (Fig. 1c), no significant morphological differences are observed. In the XPS survey scans (Fig. 1a), of pristine P50 and ZnO on P50 for binding energy range of 0–1400 eV, only a C 1s peak at ∼284 eV appears in pristine P50, while distinct Zn, O, and C peaks were detected in ZnO on P50. No trace contaminants resulting from the simple dipping and heating process are observed on the surface of ZnO on P50 sample.^27^ The elemental analysis from XPS suggests that the actual loading amount of ZnO is approximately 18 wt%.

**Fig. 2:**
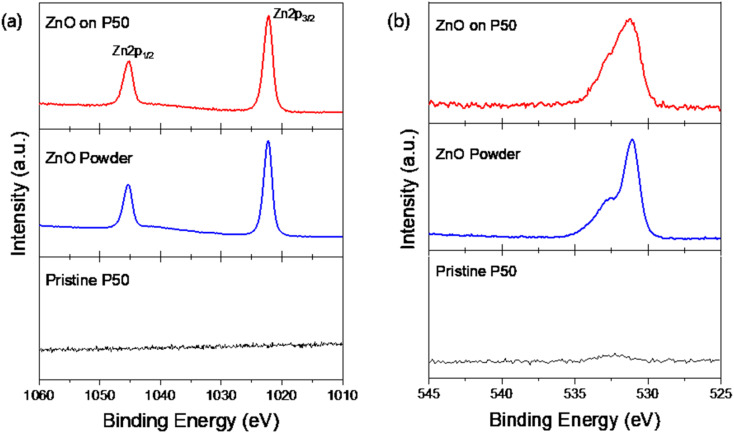
High resolution XPS spectra in the core-level region of Zn 2p (a) and O 1s (b).

**Fig. 3:**
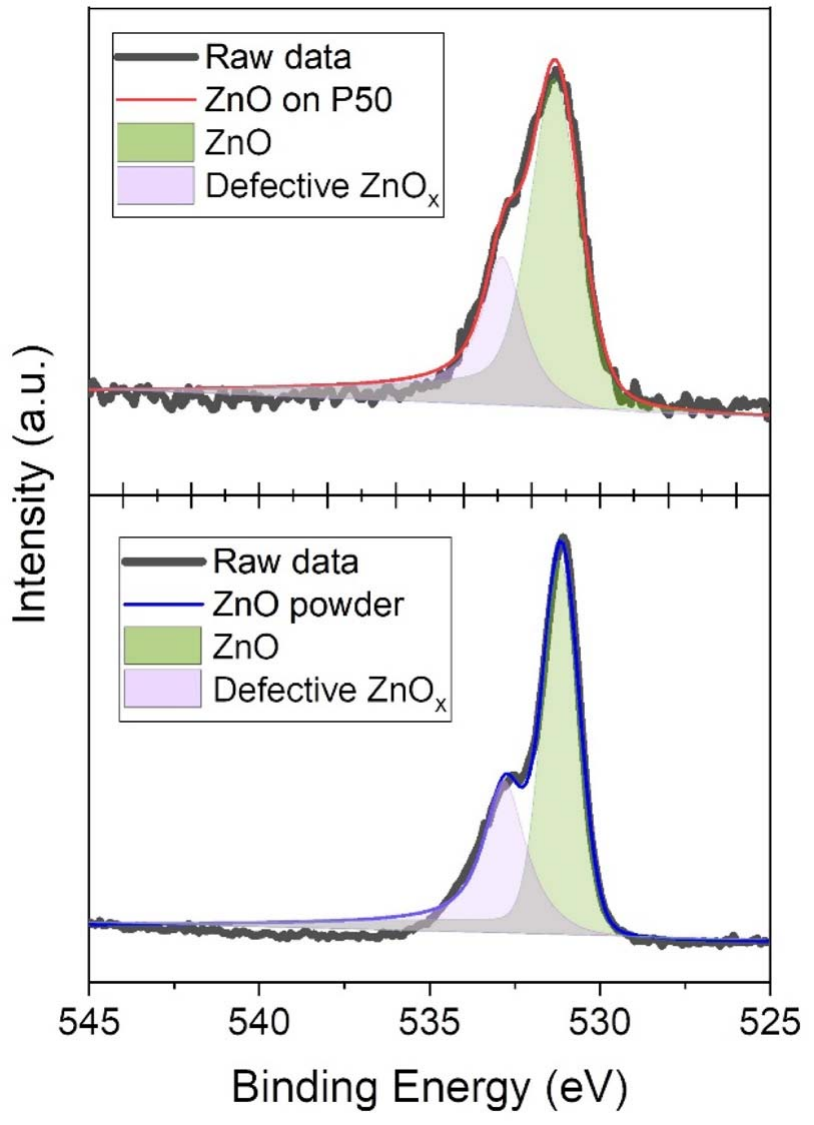
Curve-fitted O 1s spectra of ZnO on P50 and ZnO powder.

Fig. 2 shows the high-resolution XPS *spectra* of Zn 2p (Fig. 2a) and O 1s (Fig. 2b). In the range 1010–1060 eV, the Zn 2p core-level XPS spectra of ZnO on P50 (red) and ZnO powder (blue) displayed doublet splitting at approximately 1022 and 1045 eV, corresponding to Zn 2p_1/2_ and Zn 2p_3/2_ core-levels, respectively.^28^ However, no distinguishable peaks were detected for the pristine P50 (black, Fig. 2a). Similarly, the O 1s spectra for both ZnO on P50 and ZnO powder show peaks at approximately 532 eV, while the pristine P50 exhibited no corresponding peak (Fig. 2b).^29^ These results indicate that the presence of ZnO, which was not detectable in the XRD analysis of ZnO on P50 (Fig. S1), was successfully identified through the XPS survey spectrum, and a minor quantity of ZnO was integrated within the carbon network of ZnO on P50. Fig. 3 shows the curve-fitted O 1s spectra of ZnO on P50 and ZnO powders, which further demonstrates the difference in chemical composition between the two samples. The two asymmetric peaks are associated with the O_2_^−^ ions within the typical wurtzite ZnO lattice (light green) and the O_2_^−^ ions within the oxygen-deficient region (defective ZnO_*x*_, light purple) in both ZnO on P50 and ZnO powder. Oxygen vacancies, as one of the most prevalent point defects in metal oxides, have been extensively recognized for their crucial role in enhancing the electrochemical performance of lithium–air batteries. These vacancies introduce localized electronic states near the conduction band, often leading to significant modifications of the electronic structure. Specifically, oxygen-deficient sites generate defect states within the band gap, effectively narrowing the band gap and increasing the density of free carriers. As a result, the electrical conductivity of the metal oxide is markedly improved, which facilitates faster charge transfer kinetics during both the oxygen reduction reaction (ORR) and oxygen evolution reaction (OER) processes.^30^ In addition to electronic enhancements, oxygen vacancies function as catalytically active sites that strengthen the adsorption and activation of oxygen species. The under-coordinated metal centers formed adjacent to vacancy sites provide electron-rich environments that promote strong interaction with molecular oxygen (O_2_) and superoxide intermediates (O_2_^−^). This strengthened adsorption not only lowers the activation barrier for O_2_ reduction but also enables the stabilization of reactive ORR intermediates, such as LiO_2_. The stabilization of LiO_2_ plays a key role in directing the reaction pathway toward the formation of Li_2−*x*_O_2_—a non-stoichiometric, electronically conductive discharge product that offers lower overpotential and enhanced reversibility compared to stoichiometric Li_2_O_2_.^31,32^

The notable differences in chemical composition between the two samples became even clearer upon examination of the curve-fitted O 1s spectra of ZnO on P50 and ZnO powder, as illustrated in Fig. 3. The two asymmetric peaks are associated with the O_2_^−^ ions in the typical wurtzite ZnO lattice (light green) and O_2_^−^ ions located in oxygen-deficient regions within the ZnO matrix (defective ZnO_*x*_, light purple) in both ZnO on P50 and ZnO powder samples.^33,34^

**Fig. 4:**
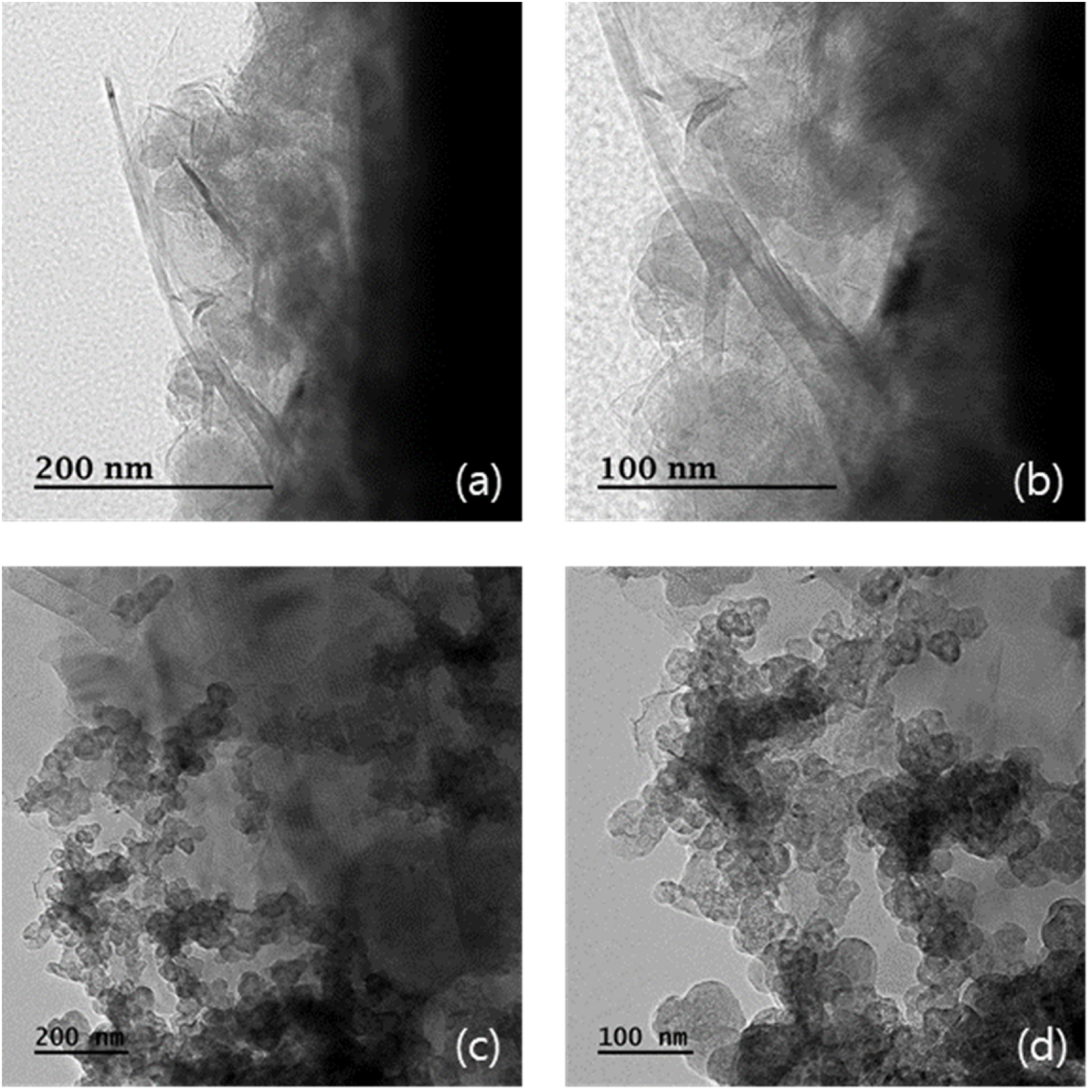
FE-TEM images of the pristine P50 (a and b) and ZnO on P50 (c and d).

The morphologies and particle structures of the prepared samples were verified using HR-TEM. Fig. 4 presents the HR-TEM images of pristine P50 (a and b) and ZnO on P50 (c and d). Amorphous features are exclusively observed in pristine P50 (Fig. 4a and b), indicating the presence of carbon without distinct particulate structures. By contrast, spherical particles are clearly visible and well dispersed within the amorphous carbon matrix in ZnO on P50, as shown in Fig. 4c and d.

**Fig. 5:**
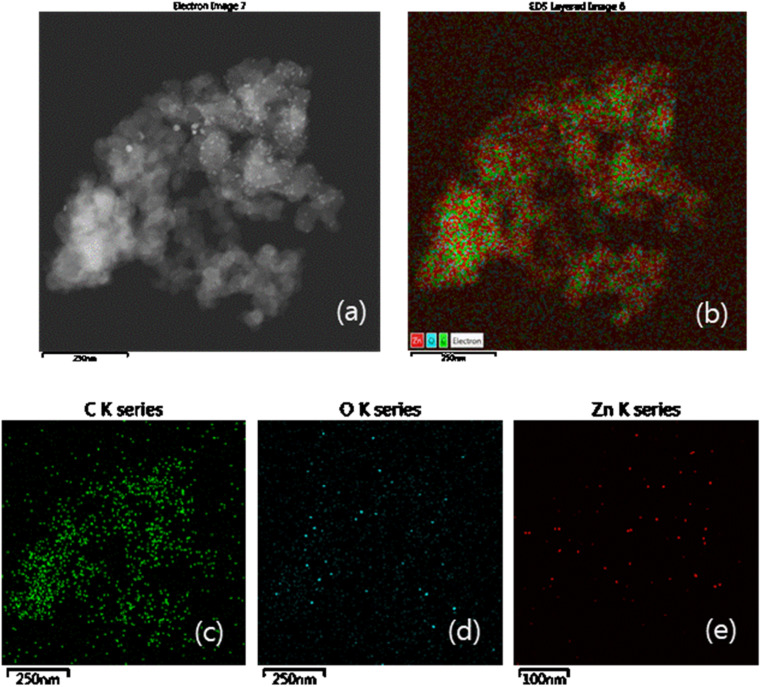
Dark field TEM image (a). EDS elemental mapping images (b) and the corresponding elemental maps for C (c), O (d) and Zn (e) of ZnO on P50.

Elemental mapping analysis clearly shows the features of ZnO on P50, in which the ZnO particles are uniformly distributed within the carbon matrix. Fig. 5 shows the dark-field (a) and elemental mapping (b) images, and the corresponding elements for C (c), O (d), and Zn (e) of ZnO on P50. The dark-field TEM image of ZnO on P50 (Fig. 5a) reveals the presence of spherical nanoparticles, which is further corroborated by EDS elemental mapping (Fig. 5b). The mapping in Fig. 5b shows that Zn (red) and O (pastel blue) are uniformly distributed across the C (green) background, and their respective elemental maps are displayed in Fig. 5c–e. This provides additional evidence that ZnO was successfully integrated into the carbon matrix.

**Fig. 6:**
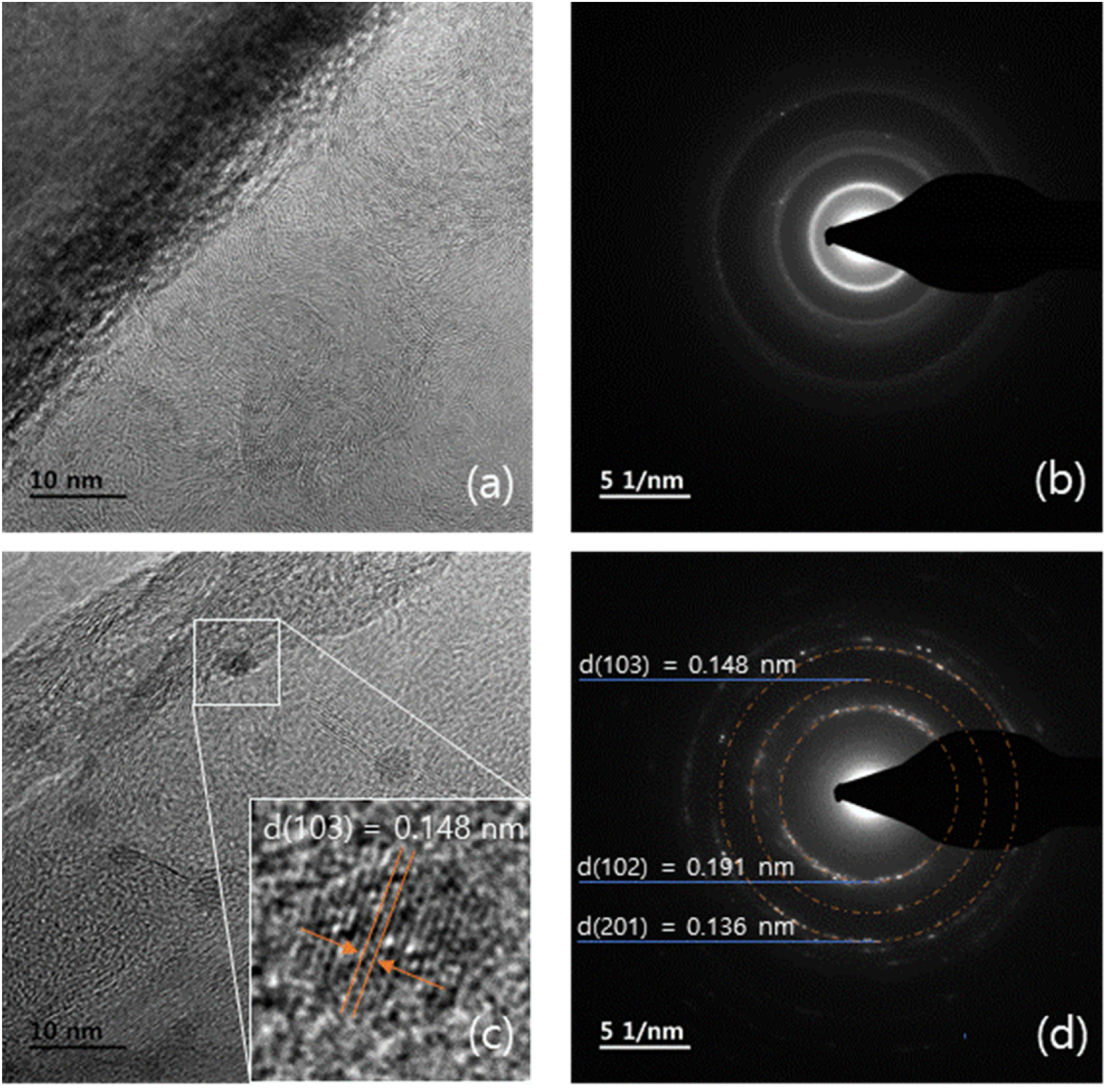
Lattice fringe of P50 (a), ZnO On P50 (c) and SAED analysis of P50 and ZnO on P50 (b and d).

Fig. 6 presents the lattice fringe images and SAED (Selected Area Electron Diffraction) patterns of the P50 and ZnO on P50 composites. Fig. 6a shows a high-resolution TEM image of P50, revealing a characteristic amorphous structure along with embedded carbon nanofibers. In the ZnO on P50 sample, ZnO crystallites are clearly observed, and the magnified inset (Fig. 6c, highlighted by the white box) displays distinct lattice fringes within the ZnO nanocrystals, along with observable structural defects attributed to oxygen vacancies. The measured *d*-spacing of the fringe is approximately 0.148 nm, corresponding to the (103) plane of wurtzite ZnO. The SAED pattern of ZnO on P50 in Fig. 6d shows a noticeably different diffraction pattern compared to pristine P50, with well-defined rings corresponding to *d*(103) = 0.148 nm, *d*(102) = 0.191 nm, and *d*(201) = 0.136 nm. These values are consistent with the (102), (103), and (201) planes of wurtzite ZnO. The presence of clear lattice fringe and diffraction rings confirms the nanocrystalline nature of ZnO containing oxygen vacancies, thereby verifying the crystallinity of the ZnO on P50 composite.

The ZnO on P50 composite was subsequently employed as the cathode material in a rechargeable non-aqueous Li–O_2_ battery. Fig. 6 shows the 1st (a), 10th (b), 20th (c), 30th (d), and 40th (e) discharge–charge profiles at a current of 0.1 mA for 5 h (limiting capacity: 0.5 mAh). Results are shown for both pristine P50 (black) and ZnO on P50 (red). The cycling performance and corresponding energy efficiencies are summarized in Fig. 6f. In the initial few cycles, there was little noticeable improvement; however, after the fifth cycle, an increase of approximately 6% was observed in energy efficiency, with stable operation maintained up to 40 cycles, as depicted in Fig. 6e.

Fig. S3 shows that the ZnO on P50 sample exhibits improved cycling stability, reaching 75 cycles compared to 50 cycles for the pristine P50. Although the cycle life of the ZnO on P50 sample is limited to fewer than 100 cycles in this study, the result primarily reflects the catalytic effect of ZnO, rather than a fully optimized system. Therefore, achieving a cycle life beyond 100 cycles with ZnO on P50 is considered feasible through further optimization in future studies.

In lithium–air battery systems, the decline in coulombic efficiency is closely related with parasitic reactions and incomplete OER (oxygen evolution reactions) that occur on the cathode surface during the charging process. These detrimental effects lead to the accumulation of residual by-products, which reduce the active surface area available in subsequent cycles. As a result, coulombic efficiency progressively decreases, accompanied by poor cycle-to-cycle reproducibility and shortened cycle life. During the initial cycles, both samples exhibit comparable coulombic efficiencies, as negligible parasitic reactions and incomplete OER occur, leaving minimal residuals. However, a significant divergence emerges after the fifth cycle. This behavior is attributed to the formation of highly conductive and low-volume discharge products generated *via* the bifunctional cathode mechanism, which effectively mitigates the accumulation of resistive by-products and preserves the electrochemical activity of the cathode surface.^35^

We tabulate the performance and synthetic complexity of other metal oxides, including ZnO, and show that ZnO exhibits superior cycle properties compared to other oxides, as well as low cost and facile synthesis method. Although our previous studies applied easy synthetic methods, catalysts such as RuO_2_ (ref. 36) and IrO_2_ (ref. 37) are expensive and require complex synthetic methods.

The catalytic effects of the ZnO on P50 catalyst on reducing overpotential and enhancing energy efficiency are understood with previous studies on bifunctional cathodes. According to the reported work by Fan *et al.* on ZnO@VACNT, O_2_ undergoes oxygen reduction reactions (ORR) on the surface of carbon nanotubes (CNTs), forming superoxide (O_2_^−^), which is then either adsorbed at the ZnO/VACNT interface or dissolved into the electrolyte.^35^ This reduced superoxide, possessing an abnormal oxidation state, facilitates the formation of non-stoichiometric, defect-rich Li_2−*x*_O_2_ discharge products, often referred to as LiO_2_-like species (Table 1).

**Table 1:** Properties of various metal oxides

Catalysts	Performance	Cost	Synthesis complexity
ZnO	40 cycles	Low	Simple (dipping & heating)
Mn_3_O_4_	40 cycles	Low	Simple (dipping & heating)
NiO	50 cycles	Medium	Simple (dipping & heating)
CuO	9 cycles	Low	Simple (dipping & heating)
RuO_2_	20 cycles	High	Complex (one-pot solvothermal reaction)
IrO_2_	10 cycles	High	Complex (CVD & hydrothermal method)

Compared to conventional Li_2_O_2_ products, Li_2−*x*_O_2_ exhibits lower overpotential during electrochemical reactions, along with higher electronic conductivity and reduced volume, resulting in significantly improved energy efficiency and energy density.^38^ Although ZnO does not serve as a direct catalyst for redox reactions, it plays a crucial role in promoting the formation of LiO_2_-like discharge products and stabilizing them by guiding the formation of nanostructured and uniformly distributed phases.

**Fig. 7:**
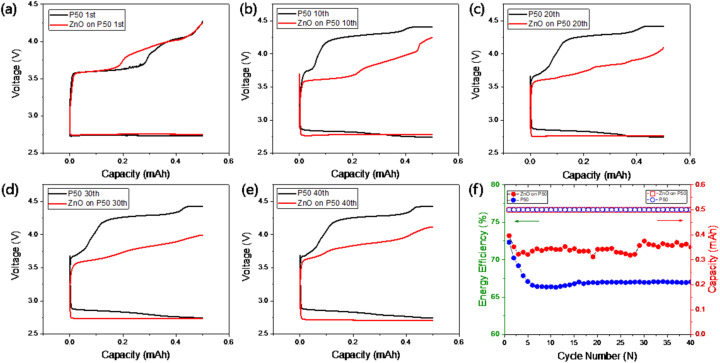
1st (a), 10th (b), 20th (c), 30th (d) and 40th (e) discharge–charge profiles of the Li–O_2_ cell using pristine P50 (black) and ZnO on P50 (red) at a current density of 0.5 mAh and their cycle performance and energy efficiency (f).

*Ex situ* XRD analysis was performed to confirm if the aprotic Li–O_2_ battery operates as per theoretical principles. Fig. 7 presents the *ex situ* XRD patterns for each corresponding state of the cell using pristine P50 (a) and ZnO on P50 (b), where the formation of Li_2_O_2_ (red asterisk) during the discharge state and its disappearance upon the fully recharged state can be observed in both samples, excluding the expected undesirable byproducts. The LiOH (green inverted triangle) observed in the recharged state of the pristine P50 sample is believed to have formed during the XRD measurement process after washing, while the Li_2_CO_3_ (blue circle) detected in the discharged state of the ZnO on P50 possibly resulting from electrolyte decomposition induced by the catalyst. The O_2_^−^ (superoxide) species generated on the surface or interface of defective ZnO contribute to the stabilization of LiO_2_ intermediates, thereby enhancing the electrochemical performance of lithium–air batteries. However, the same reactive oxygen species participate in side reactions at the carbon electrode–electrolyte interface, leading to the formation of undesirable byproducts such as Li_2_CO_3_.^39^ Li_2_CO_3_ is a highly irreversible byproduct that is difficult to decompose even at charging potentials above 4.38 V. Its continuous accumulation on the electrode surface leads to a significant reduction in active surface area, ultimately resulting in decreased coulombic efficiency and shortened cycle life. To address these challenges, several strategies have been proposed: (1) introducing redox-active species as charge mediators to compensate for electronic losses during charging, and (2) employing oxygen-selective membranes on the cathode to prevent the permeation of CO_2_ and H_2_O, thereby suppressing the formation of Li_2_CO_3_.^31,32^ These approaches, if necessary, will be discussed in more detail in future studies.^40^

**Fig. 8:**
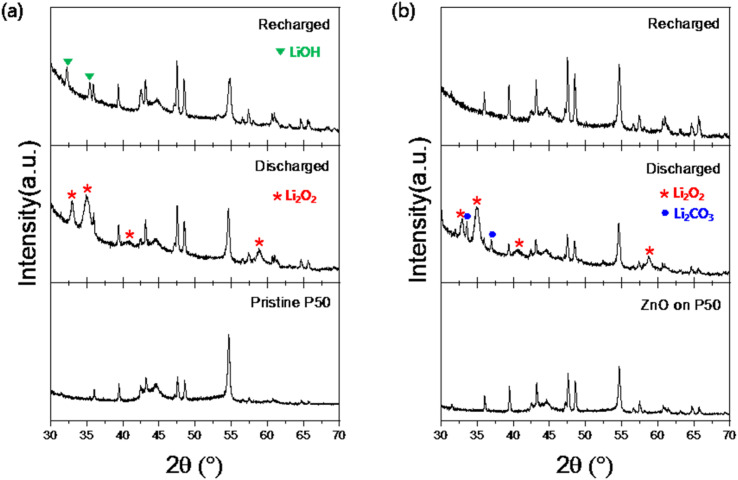
*Ex situ* XRD patterns at different state of the Li–O_2_ cell using pristine P50 (a) and ZnO on P50 (b).

Fig. 8 shows SEM images of the discharge products (Li_2_O_2_). In the case of pristine P50 (Fig. 8a), agglomerated Li_2_O_2_ particles were observed. By contrast, the ZnO on P50 sample (Fig. 8b) exhibited a more uniform and finely distributed morphology. The uniformity of Li_2_O_2_ in ZnO on P50 is attributed to the well-defined active sites provided by ZnO, which were introduced into the carbon through an *in situ* direct growth method, thereby exhibiting a catalytic effect on the electrochemical properties of the aprotic Li–O_2_ cell. The hierarchical mesoporous structure is composed of porous structures inside and outside the nanocage, providing diffusion channels for Li^+^/O_2_. In addition, ZZFC has a rational structure that combines the characteristics of large surface area and uniformly distributed active sites, which is advantageous for mass/electron transfer in multiphase discharge/charge reactions. It was reported that the TEGDME-based electrolyte is stable with ZZFC during operation, and the parasitic reaction is reduced, resulting in reversible generation/decomposition of Li_2_O_2_ as a discharge product.^16^

**Fig. 9:**
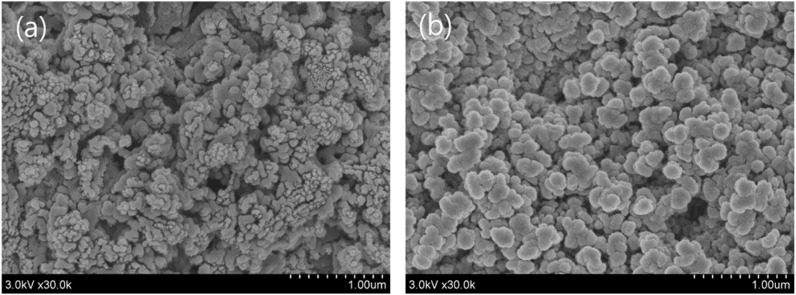
SEM images of discharge product (Li_2_O_2_): pristine P50 (a), ZnO on P50 (b).

An ultra-low charge potential of 2.8 V was achieved by ALD-assisted ZnO passivation and Pd nanoparticle deposition, which is reported to be because the ALD ZnO thin films partially cover the carbon surface and selectively coat the carbon defect sites, which helps to minimize side reactions such as electrolyte decomposition and lithium carbonate formation at the defect sites. Previous density functional theory (DFT) calculations have shown that the small Al_2_O_3_ islands fabricated by ALD are small enough to be conductive by themselves, and in this case, a material more conductive than Al_2_O_3_, such as ZnO, is reported to be helpful for increasing the overall conductivity of the carbon anode when used to passivate the carbon defect sites (Fig. 9).^15^

## Conclusion

A ZnO electrocatalyst integrated onto carbon paper (ZnO on P50) was synthesized by a simple dipping of P50 carbon paper in a zinc acetate/ethylene glycol solution followed by heat treatment at 400 °C. The resulting ZnO on P50 was utilized as a cathode material for non-aqueous rechargeable Li–O_2_ batteries. The Li–O_2_ cell incorporating the ZnO on P50 cathode demonstrated an increase in energy efficiency of approximately 6.3% over 40 charge–discharge cycles.

## Conflicts of interest

The authors declare no competing interests.

## Supplementary Material

RA-015-D5RA03545G-s001

## Data Availability

The data used to support the findings of this study are included within the article. The supplementary Information contain the results of XRD(ZnO on P50, P50, ZnO powder) and cycle ability(ZnO on P50, P50). See DOI: https://doi.org/10.1039/d5ra03545g.
