# Supplementary material for: ZnO electrocatalyst integrated onto carbon paper for efficient non-aqueous Li–O2 batteries
Source: RSC Adv. 2025 Aug 11;15(35):28348–57. doi: 10.1039/d5ra03545g (PMC12377233; doi:10.1039/d5ra03545g)
Supplement: RA-015-D5RA03545G-s001 [file RA-015-D5RA03545G-s001.pdf]

Supplementary Information (SI) for RSC Advances.
This journal is © The Royal Society of Chemistry 2025

ZnO electrocatalyst integrated onto carbon paper for efficient non-aqueous Li−O_2 batteries

Inhan Kang, Sehun Kim, Su Min Lee, Se Hyun Jeong, Hyeong Ju Ki, Ui Jin Lee, Jae Hyung Ko, Byung Jun Son, Sungjin Kim* and Jungwon Kang*

Department of Advanced Materials Science and Engineering, Mokpo National University, 61 dorim-ri, 1666 Yeongsan-ro, Cheonggye-myeon, Muan-gun, Jeonnam, South Korea

*Corresponding author: ksj840711@gmail.com, jwkang17@mokpo.ac.kr

Supplementary Information

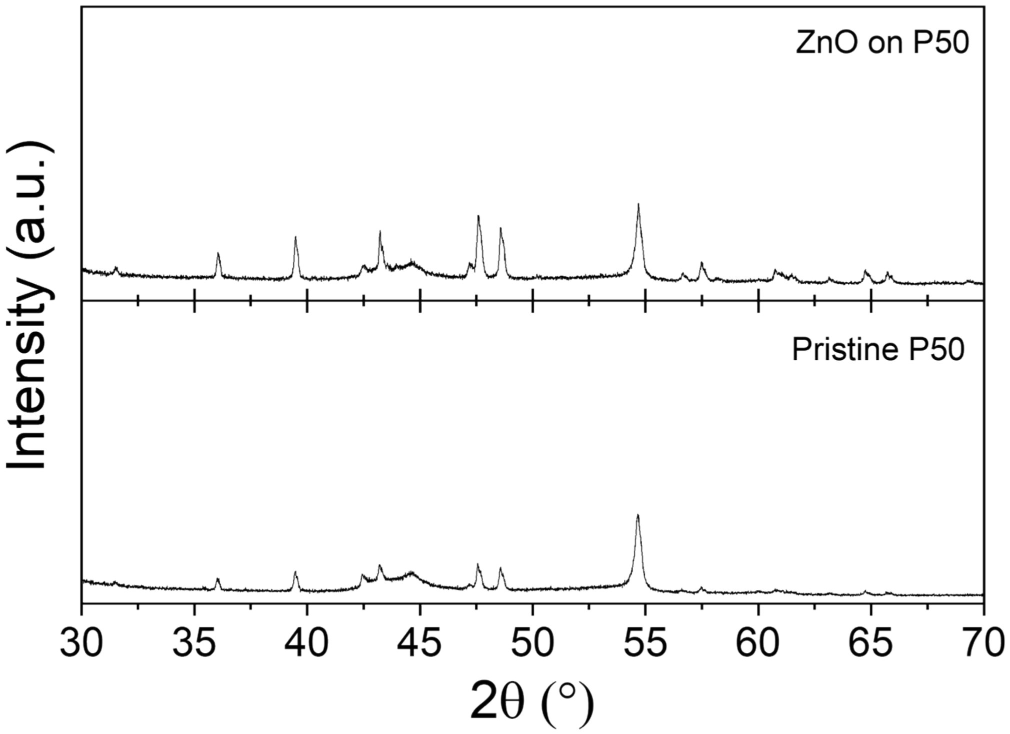

Figure S1. XRD patterns of ZnO on P50 and pristine P50

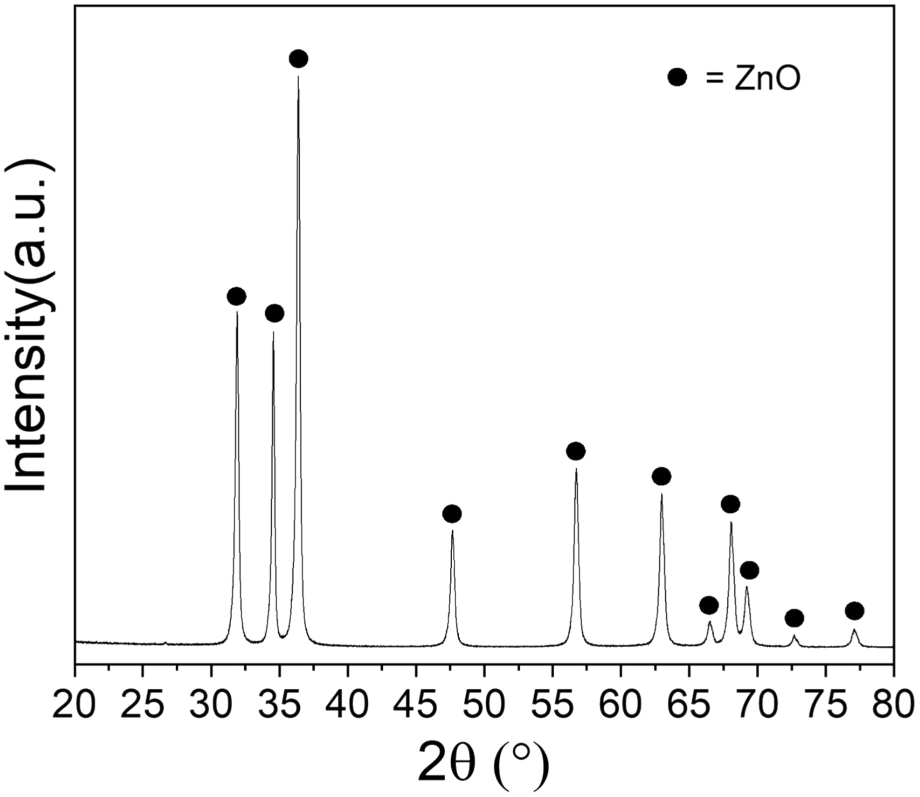

Figure S2. XRD patterns of ZnO powder (zinc acetate dihydrate powder heated at 400 ℃ in air)

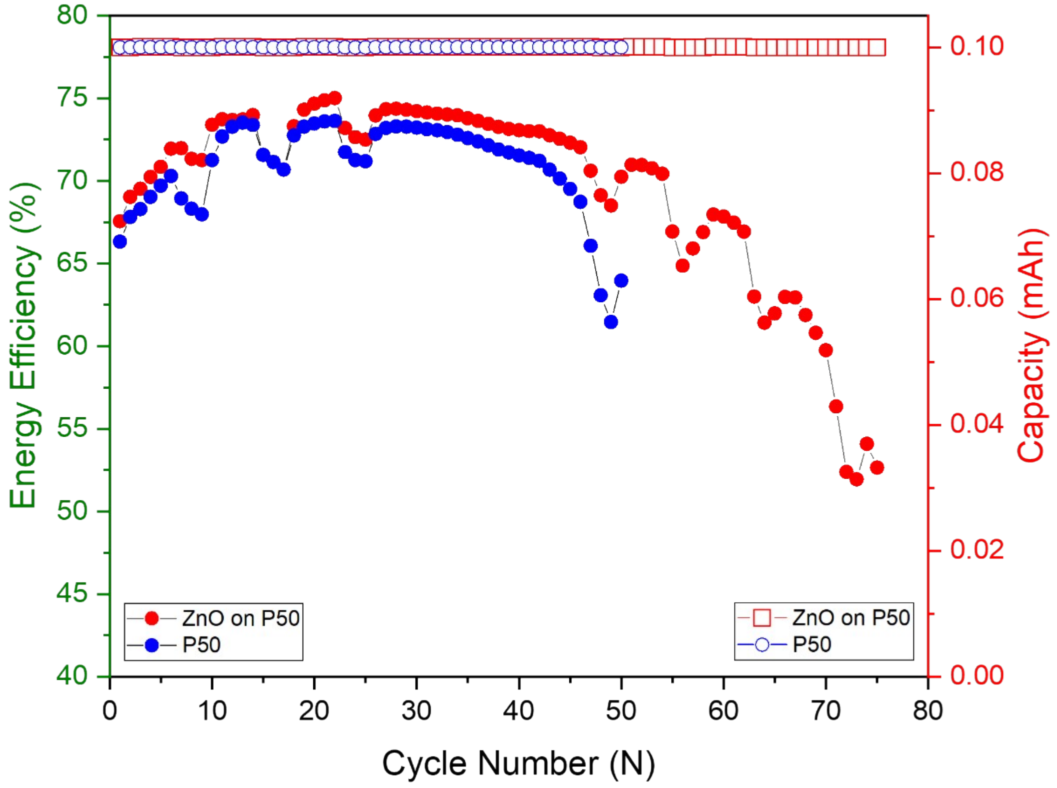

Figure S3. Energy Efficiency of P50 and ZnO on P50 at a current density of 0.1 mAh
